# Adropin Serum Levels in Patients with Primary Sjögren’s Syndrome

**DOI:** 10.3390/biom11091296

**Published:** 2021-08-31

**Authors:** Marijana Janković Danolić, Dijana Perković, Marin Petrić, Igor Barišić, Katarina Gugo, Joško Božić

**Affiliations:** 1 School of Medicine, University of Split, 21000 Split, Croatia; 2 Department of Internal Medicine, Division of Clinical Immunology and Rheumatology, University Hospital of Split, 21000 Split, Croatia; dijana.perkovic@hotmail.com (D.P.); petrinjo19@gmail.com (M.P.); 3 Department of Diagnostic and Interventional Radiology, University Hospital of Split, 21000 Split, Croatia; igorbarisc@net.hr; 4 Department of Medical Laboratory Diagnostics, University Hospital of Split, 21000 Split, Croatia; katarina.gugo@gmail.com; 5 Department of Pathophysiology, University of Split School of Medicine, 21000 Split, Croatia; josko.bozic@mefst.hr

**Keywords:** primary Sjögren’s syndrome, adropin, endothelial dysfunction, anti SSA/Ro52 antibodies, SSDDI

## Abstract

Primary Sjögren’s syndrome (pSS) patients have higher prevalence of endothelial dysfunction and premature atherosclerosis. Recent studies investigated adropin, a secretory protein that can regulate lipid metabolism and insulin resistance and protect endothelial cells’ function and that has an anti-inflammatory effect. The aim of this study was to determine adropin levels in pSS patients compared to healthy controls. Additional goals were exploring the correlation between adropin and several metabolic and immunological parameters in pSS, including disease specific antibodies, EULAR Sjögren’s Syndrome Disease Activity Index (ESSDAI), and Sjögren’s Syndrome Disease Damage Index (SSDDI). This research included 52 pSS patients and 52 healthy controls. pSS patients have significantly higher adropin levels compared to the control group (3.76 ± 0.68 vs. 3.14 ± 0.69 ng/mL, *p* < 0.001). Correlation analysis showed that adropin levels in pSS patients have positive correlation with high-density lipoprotein (HDL) (r = 0.290, *p* = 0.036) and anti SSA/Ro52 antibodies (r = 0.307, *p* = 0.026) and negative correlation with SSDDI (r = −0.401, *p* = 0.003). Multivariant linear regression showed that adropin levels are independently associated with HDL (β ± SE, 0.903 ± 0.283, *p* = 0.002) and SSDDI (β ± SE, −0.202 ± 0.073, *p* = 0.008). Our findings imply that adropin could be involved in the pathophysiology of pSS, yet it remains to be elucidated in future studies whether adropin has a protective or detrimental role in this setting.

## 1. Introduction

Sjögren’s syndrome (SS) is a chronic systemic autoimmune disease that is characterized by lymphocytic infiltration of the exocrine glands, resulting in symptoms of dry eyes and dry mouth, and objective evidence of xerophthalmia and xerostomia. SS can present as an entity by itself, without an underlying autoimmune condition—primary SS (pSS)—or may occur with an underlying autoimmune condition—secondary SS (sSS) [1]. There are studies showing that traditional cardiovascular (CV) risk factors such as hypertension (AH) and dyslipidemia appear with increased rates in pSS [2]. While the CV incidence and survival rates are not fully known in pSS patients, they are still listed among the most important causes of mortality in this population [3]. Comparably to the other autoimmune diseases such as systemic lupus erythematosus (SLE) and rheumatoid arthritis (RA), subclinical atherosclerosis is also frequently observed among population with pSS [4,5,6,7]. Recent investigations indicate that lower adropin levels suggest higher CV risk. However, there is a shortage of available evidence regarding the association of adropin and autoimmune diseases [8,9].

Adropin is a newly identified secretory protein that participates in the regulation of energy homeostasis and insulin response. It is encoded by the energy homeostasis associated gene (ENHO) and expressed in the liver, brain, heart, and coronary endothelial cells [10,11,12]. It was first identified in 2008 by Kumar et al. during microarray analysis of the liver gene expression in mouse models of obesity [10]. Moreover, adropin appears to participate in the maintenance of energy homeostasis and insulin response, closely related to the development and progression of atherogenesis [10]. Lower adropin levels lead to endothelial impairment and dysfunction [11]. Consequently, decreased serum adropin levels weaken endothelial protection and may cause or accelerate atherosclerosis [8,13,14,15].

The main goal of our study was to compare the levels of adropin in patients with primary Sjögren’s syndrome and healthy controls. In addition, we aimed to establish a connection between adropin and several metabolic and immunological parameters in pSS, including Sjögren’s syndrome specific antibodies, disease activity, and damage scores.

## 2. Materials and Methods

### 2.1. Participants and Study Design

This cross-sectional case-control study was performed on 52 patients with pSS treated at Department of Rheumatology and Clinical Immunology, University Hospital of Split, University of Split, School of Medicine, between January 2018 and February 2019. All patients fulfilled the American–European Consensus Group criteria for the classification of primary SS [16]. The inclusion criteria for patients were that they were above 18 years of age and had had a diagnosis of pSS for more than a year. Exclusion criteria were a history of other inflammatory diseases (RA, SLE, vasculitis), diabetes mellitus, chronic renal failure, essential mixed cryoglobulinemia, IgG4 syndrome, hematopoietic malignancies, or overt cardiovascular disease (acute myocardial infraction, angina pectoris, stroke, or peripheral arterial disease). The control group included 52 sex, age, smoking, and body mass index (BMI)-matched healthy subjects.

### 2.2. Ethical Considerations

All patients signed written informed consent before enrollment in the study upon being informed about the procedures, course, and purpose of the study. The study was approved by the Ethics Committee of University Hospital of Split (date of approval: 25/10/2017) and University of Split School of Medicine (date of approval: 27/10/2017), and it was conducted in accordance with all ethical principles of the Seventh Revision of the Helsinki Declaration from 2013.

### 2.3. Clinical and Laboratory Evaluation

Participants were evaluated using a standardized clinical interview. They were assessed for sociodemographic, anthropometric, and clinical data; traditional CV risk factors; comorbidities; and current medications. The patients’ clinical data (xerostomia, xeropthalmia), disease duration, Schirmer’s test, and biopsy findings were obtained from the hospital notes and electronic patient records. After detailed physical examination, blood samples were taken. Fasting blood specimens for biochemical and immunologic tests were analyzed by an experienced blinded medical biochemist and routinely handled according to the standard laboratory practice by the central laboratory of our center. Standard laboratory parameters, complement components, and adropin were determined in pSS patients and controls. Autoantibodies were determined only in patients with pSS.

The samples for analyses of serum adropin levels were centrifuged and stored at −80 °C for further analyses, while the hematological and biochemical parameters were analyzed on the same day using the standard laboratory procedures. Serum adropin levels were determined using the dual enzyme-linked immunosorbent assay (ELISA) of human adropin (Phoenix Pharmaceuticals, Burlingame, CA, USA) according to the manufacturer’s instructions. Calibrations were double measured, whereas optical density (OD) values were in accordance with the predefined OD values stated in the manufacturer instructions, and the coefficient of variability of paired calibrations was <15%. The linear range of the assay was 0.3–8.2 ng/mL, and sensitivity was 0.3 ng/mL, while the coefficient of variability within the probe was less than 10%.

Antinuclear antibodies (ANA) were detected using indirect immunofluorescence assay on HEp-2 cells substrate (Inova Diagnostics, San Diego, CA, USA). Results of ANA IFA were reported with titre and fluorescence pattern. Cut-off titre for positive results was set to 1:160 and patients positive at titre 1:80 were reported as borderline positives. Specific antibodies including anti SSA/Ro60, anti SSA/Ro52, and anti SSB/La were measured using Fidis Connective Profile 14 test (Theradiag, Marne-la-Vallée, France) on a Luminex 100/200 analyzer. This semi-quantitative addressable laser bead immunoassay enables simultaneous detection of 14 different ANA specific antibodies with distinction between anti SSA/Ro60 and anti SSA/Ro52 antibodies. ANA and anti SSA/Ro 60, anti SSA/Ro 52, and anti SSB/La were analyzed only in pSS group. Other biochemical parameters were analyzed according to standard laboratory procedures.

### 2.4. Definition of CV Risk

Blood pressure (BP) was measured twice, separated by 5 min, on the dominant arm, with the subject in a seated position after at least 5 min of rest, and the mean was calculated to achieve a more precise value. BP was taken by the same physician using a validated automatic oscillometric device (Rudolf Riester GmbH, Jungingen, Germany). Hypertension was defined as systolic BP ≥ 140 mm Hg, diastolic BP ≥ 90 mm Hg, or normal BP values in patients receiving antihypertensive treatment. Hypercholesterolemia was defined as total cholesterol (TC) > 5.0 mmoL/L or receiving statins, whereas triglycerides (TG) > 1.7 mmoL/L indicated hypertriglyceridemia. Body height and weight were determined using a medical scale with built-in heights (Seca, Birmingham, UK). Body mass index (BMI) was calculated using the formula = body mass/height^2^ (kg/m^2^). Obesity was defined as body mass index (BMI) ≥ 30 kg/m^2^. A participant was considered a smoker if they had smoked at least one cigarette per day during the year before inclusion. Low physical activity was defined as <3 days/week of at least 45 min of moderately intense aerobic physical exercise. Menopause status was considered if >1 year had passed since the last menstrual period. A Framingham risk score (FRS) was derived for each subject using the gender-specific prediction formulae proposed by Wilson et al. based on conventional CV risk factors (age, TC and HDL cholesterol blood pressure, diabetes and smoking status) [17]. For this study, it was automatically calculated using the calculator from website: https://www.mdcalc.com/framingham-risk-score-hard-coronary-heart-disease (accessed on 26 May 2018).

### 2.5. Evaluation of the Activity and Accumulated Irreversible Damage in Primary SS

Primary SS activity was measured by means of the EULAR Sjögren’s Syndrome Disease Activity Index (ESSDAI), which includes 12 domains: constitutional, lymphadenopathy, glandular, articular, cutaneous, pulmonary, renal, muscular, peripheral nervous system, central nervous system, hematologic, and biologic [18]. Additionally, the accumulated irreversible damage in primary SS was evaluated by means of the Sjögren’s Syndrome Disease Damage Index (SSDDI), which comprises the following domains: ocular, oral, and systemic (neurologic, renal, pulmonary, cardiovascular, gastrointestinal, musculoskeletal, endocrine, and malignancy) damage [19]. ESSDAI and SSDDI were evaluated by licensed rheumatologist.

### 2.6. Statistical Analysis

Statistical analysis performed using MedCalc package, version 19.1.2 (MedCalc Software, Ostend, Belgium). Categorical variables were expressed as whole numbers (N) and percentages (%) and continuous variables as mean and standard deviation, or median and interquartile range, according to normality of data distribution which was assessed with the Kolmogorov-Smirnov test. Accordingly, statistical differences between categorical variables were determined with the Chi-squared test, while differences between continuous variables were determined with the *t*-test for independent samples and the Mann-Whitney U test. Furthermore, correlations between adropin and other parameters were assessed with Pearson’s and Spearman’s correlation coefficients. Finally, factors independently associated with adropin levels were determined using multiple linear regression analysis. Unstandardized beta coefficients (β), standard error (SE), *t* and *p*-values were reported. Statistical significance was set at *p* < 0.05.

Sample size was determined with MedCalc statistical package as well, using *t*-test for independent samples. In a pilot study on 15 randomly selected participants with pSS and 15 control participants, adropin levels were assessed. The difference between two means of adropin levels was 0.43 and standard deviation was 0.59. With α error set as 0.05, and β error of 0.1, the calculated required sample size was 40 subjects per group.

## 3. Results

### 3.1. Baseline Characteristics of the Study Population

We included 52 patients with pSS and 52 healthy control subjects matched by sex, age, smoking, and BMI. There were no statistically significant differences in age, sex, or anthropometric features between the pSS patients and the control group (*p* > 0.05; for all analysis) (Table 1). The mean age of patients with pSS was 59.3 ± 11.1 vs. 57.8 ± 10.5 years for control group. Patients had stable disease, with median of activity index score, ESSDAI 2 (IQR 1–3), median of disease specific damage score, SSDDI 2 (IQR 2–3), and a median of disease duration of 6 years 6 (IQR 3.5–10). SBP and DBP were significantly higher in patients with pSS compared with controls (126.2 ± 16.8 vs.118.9 ± 13.2 mmHg, *p* = 0.015 and 81.4 ± 7.6 vs. 77.6 ± 8.1 mmHg, *p* = 0.014) (Table 1). Patients had higher 10-years risk for CV disease 3.4 (IQR 1.3–5.5) vs. 2.1 (IQR 1.0–3.3), *p* = 0.042, although almost all participants were classified at low 10-year risk to the FRS.

### 3.2. Laboratory Parameters of the Study Population

The pSS group had significantly lower levels of hemoglobin (*p* < 0.001) and leukocytes (*p* = 0.001) and a higher erythrocyte sedimentation rate [ESR (*p* = 0.006)], while the control group had significantly higher levels of TC (*p* = 0.010). There were no statistically significant differences between the pSS group and the control group regarding the other parameters. (Table 2). Anti SSB/La antibodies were negative in 25 pSS patients (48.1%), positive in 23 (44.2%), and marginal in 4 (7.7%) pSS patients. Anti SSA/Ro52 antibodies were negative in 15 patients (28.8%) and positive in 37 (71.2%) pSS patients. Anti SSA/Ro60 antibodies were negative in 11 (21.2%) and positive in 41 (78.8%) pSS patients.

### 3.3. Serum Adropin Levels in Patients with pSS and Control Subjects

**Figure 1:**
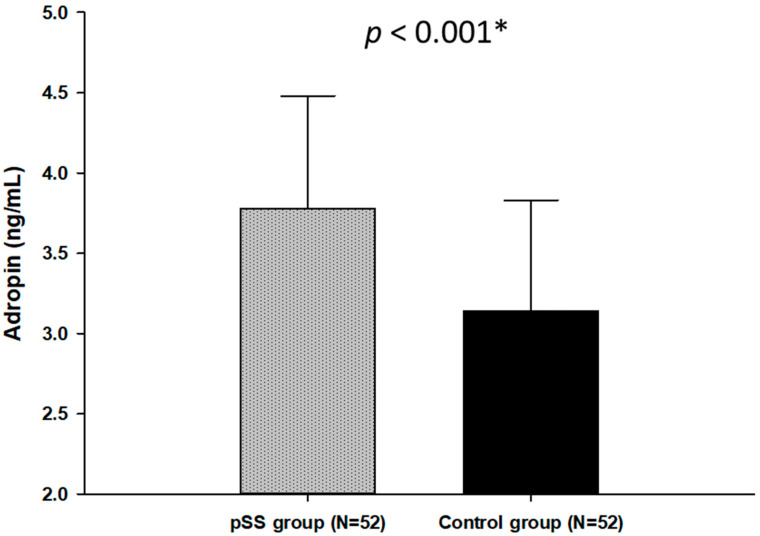
Plasma adropin levels in the pSS group and the control group. Data are presented as mean ± standard deviation. * *t*-test for independent samples.

Serum adropin levels were significantly higher in patients with pSS in comparison with the healthy control group (3.76 ± 0.68 vs. 3.14 ± 0.69 ng/mL, *p* < 0.001) (Figure 1). Furthermore, after dividing the pSS patients into lower adropin (<3.73 ng/mL) and higher adropin groups (>3.73 ng/mL) according to the median value of the pSS group, there was a statistically significant difference in SSDDI score: 3.0 (2.0–4.0) vs. 2.0 (1.0–2.0), *p* < 0.001 The pSS group with reduced levels of adropin (<3.73 ng/mL) had higher SSDDI score (Table 3).

### 3.4. Correlation between Adropin and Other Parameters

**Figure 2:**
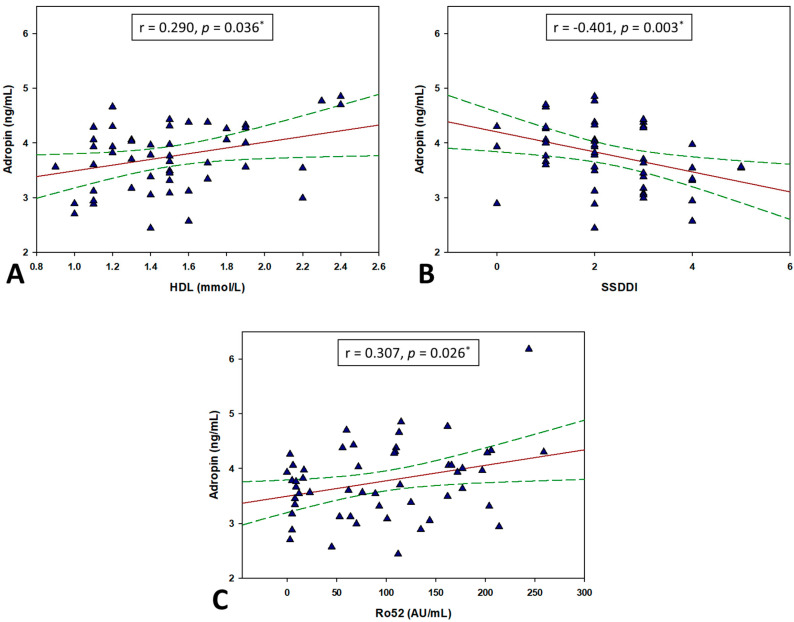
Correlation analysis of adropin levels with HDL (**A**), SSDDI (**B**) and antiSSA/Ro52 (**C**) in total study population (N = 104). * Pearson’s correlation coefficient. Red lines represent Pearson correlation coefficient and green lines represent respective 95% confidence intervals.

Correlation analysis showed that adropin levels in patients with pSS have significant positive correlation with HDL (r = 0.290, *p* = 0.036) and anti SSA/Ro52 antibodies (r = 0.307, *p* = 0.026). Statistically significant negative correlation was found with SSDDI (r = −0.401, *p* = 0.003) (Table 4) (Figure 2).

Moreover, multiple linear regression analysis showed that adropin levels retained significant association with HDL (β ± SE, 0.903 ± 0.283, *p* = 0.002) and SSDDI (β ± SE, −0.202 ± 0.073, *p* = 0.008) after model adjustment for age, BMI, and sex, with serum adropin levels as a dependent variable (Table 5).

## 4. Discussion

To our knowledge, this is the first clinical study that investigated serum adropin levels in patients with pSS. Our study reported that pSS patients have significantly higher serum adropin levels compared to a healthy control group. Furthermore, it showed that adropin levels in patients with pSS have significant positive correlation with HDL and anti SSA/Ro52 antibodies and negative correlation with SSDDI.

It is known that adropin participates in the regulation of energy homeostasis and insulin response, closely related to the development and progression of atherogenesis, having the positive effect on endothelial dysfunction [10,11,12]. Nitric oxide (NO), a potent endogenous vasodilator formed in the endothelium by the endothelial isoform of NO synthase (eNOS), plays an important role in maintaining endothelial homeostasis, preventing atherosclerotic and thrombotic processes triggered by endothelial dysfunction [20,21,22]. Accumulating evidence suggests that NO production is elevated in patients with pSS [23]. Specifically, activation of neuronal and eNOS has been described in these patients, and multiple authors have discussed how this activation is a result of increased production of antibodies against muscarinic acetylcholine receptors [24,25,26]. However, as adropin could enhance the expression of eNOS, increasing the NO bioavailability that is responsible for the production of NO in the endothelium, it is possible that adropin could at least in part explain the excessive no production observed in pSS [27,28]. Lovren et al. showed adropin can exert protective effects on the endothelial function likely mediated via upregulation of eNOS expression through the vascular endothelial growth factor receptors 2 (VEGFR2) phosphatidylinositol 3-kinase-Akt and VEGFR2-extracellular signal regulated kinase 1/2 pathways [11]. Adropin is also reported to promote critical endothelial cell function such as proliferation, migration, capillary-like tube formation, and diminished permeability. Finally, adropin decreases mRNA expression of pro-inflammatory cytokines, such as TNF-alpha and interleukin 6 (IL 6), lowering the inflammation [29]. Despite favorable effects of adropin on cardiovascular health, data suggest that increased levels of NO can have a detrimental effect upon the functioning of salivary and lacrimal glands [30].

Regarding the existing studies that connected adropin and autoimmune disease, Yolbas et al. showed no significant difference in adropin serum levels among the patients with RA and SLE and healthy controls. ENHO gene expression was significantly higher in the RA group when compared to the healthy control group, but not in terms of serum adropin levels [30]. Furthermore, increased serum adropin level were observed in a cohort of systemic sclerosis (SSc) and Behcet’s disease (BD) patients compared to the controls. These results may suggest that adropin levels increase in SSc and BD due to inflammatory processes that occur [31,32]. These finding are in line with our study results.

The most recent studies are connecting adropin with chronic inflammatory states and are proposing a possible immunomodulatory effect. It has been showed that patients with obstructive sleep apnea, inflammatory bowel diseases, polycystic ovary syndrome, and diabetes have significantly lower serum levels of adropin [33,34,35,36].

In our study adropin showed significant positive correlation with anti SSA/Ro52 antibodies. Anti SSA/Ro and anti SSB/La antibodies were closely associated with the main clinical, histopathological, and immunological features of pSS. Anti SSA/Ro52 autoantibody testing may help to identify a specific subset of SS patients with more aggressive disease and the risk of certain systemic manifestations of the disease [37]. Positive correlation of anti SSA/Ro52 antibodies and adropin in our study is in accordance with some existing research that has shown that anti SSA/Ro positive patients had lower rates of hypertension, hypercholesterolemia, and hypertriglyceridemia compared to anti-SSA/Ro and SSB/La negative patients [38]. On the other hand, Vaudo et al. have shown a significant association of anti SSA/Ro antibodies with arterial thickening [4] as an indicator of subclinical atherosclerosis. However, the role of these antibodies as well as adropin in the process of atherogenesis in these patients is controversial and requires further research.

Rather interestingly, we found significant negative correlation with the SSDDI score. Moreover, multivariant linear regression showed that the SSDDI score is also strong predictor for adropin serum levels. As a novel finding, we found that cumulative damage measured by means of the SSDDI was independently associated with decreased adropin in pSS. When pSS patients were divided according to the value of adropin into two groups, those with lower adropin serum levels had higher SSDDI scores. Cumulative damage reflects the effect of more severe disease over time, and higher SSDDI scores may identify those patients that have had more systemic inflammation and greater immunologic disturbances in the past and that most likely needed more intensive treatment, factors that are all implicated in the development of atherosclerosis [39,40]. These findings may suggest the protective anti-inflammatory role of adropin in the beginning phases and first years of disease. The fact that disease activity (ESSDAI) was not correlated with adropin was expected, since the majority of our patients had stable disease. In pSS, only two studies evaluated disease activity by ESSDAI and did not find correlation with subclinical cardiovascular damage, which is consistent with our results [41,42].

Patients with pSS had higher systolic and diastolic blood pressure and higher 10-year risk for cardiovascular events according to the Framingham calculations compared with healthy controls, but both patients and controls were classified as having a low 10-year risk according to the FRS. Several studies assessed the relationship between adropin levels and blood pressure. Gu et al. reported lower levels of adropin in adults with hypertension [43]. Low levels of adropin in hypertensive patients were also reported by Gulen et al. [44]. In contrast, Celik et al. showed that hypertensive patients have high levels of adropin [45]. It is also interesting that our pSS patients had lower levels of TC compared to the healthy control group. This is in line with the available data which highlights the influence of adropin on lipid homeostasis.

In hemodialysis patients, it was found that adropin has negative correlation with TG, LDL, and TC, while it has a significant positive correlation with HDL cholesterol [46]. The same correlations between lipids and adropin were found in several other studies [8,10,47,48]. Our study did not confirm correlation between serum adropin levels and TC, but our pSS patients had lower levels of TC in comparation with healthy controls. In our patients with pSS, serum adropin levels have significant positive correlation with HDL cholesterol. Furthermore, when multivariant linear regression was performed, HDL cholesterol was identified as an independent predictor for adropin levels, which confirms its effect in maintaining lipid homeostasis. Namely, an animal study by Akcilar et al. determined that intraperitoneal administration of low dose adropin to hyperlipidemic rats was extremely effective in decreasing the levels of serum TG, TC, and LDL cholesterol and increasing the levels of HDL cholesterol [32].

There are several limitations in the present study. Firstly, a relatively small sample size may affect the results. However, it should be taken into consideration that the population of patients diagnosed with primary Sjögren’s syndrome is limited. Furthermore, the study was conducted in a single clinical center. Finally, the cross-sectional design of the study prevents us from making inferences.

## 5. Conclusions

In conclusion, this is the first study that reported increased serum adropin levels in patients with primary Sjögren’s syndrome and demonstrated negative correlation between adropin levels and pSS damage score. These findings suggest that the augmented release of adropin may be involved in the pathogenesis of pSS, but it remains elusive whether adropin has a protective or detrimental role in this setting. Nevertheless, further large-scale studies are warranted in order to establish the precise role of adropin in pSS.

## Figures and Tables

**Table 1:** Baseline characteristics of pSS patients and healthy controls.

Parameter	pSS Group (N = 52)	Control Group (N = 52)	p *
Male sex (N, %)	4 (7.7)	3 (5.8)	0.718
Age (years)	59.3 ± 11.1	57.8 ± 10.5	0.477
Body weight (kg)	70.9 ± 11.6	71.9 ± 10.1	0.654
Body height (cm)	168.8 ± 7.1	168.3 ± 6.8	0.715
Body mass index (kg/m^2^)	24.8 ± 3.4	25.4 ± 3.1	0.418
SBP (mmHg)	126.2 ± 16.8	118.9 ± 13.2	0.015
DBP (mmHg)	81.4 ± 7.6	77.6 ± 8.1	0.014
FRS (%)	3.4 (1.3–5.5)	2.1 (1.0–3.3)	0.042
Smoking (N, %)	9 (17.3)	8 (15.4)	0.792
Disease duration (years) ^†^	6 (3.5–10)	-	-
ESSDAI	2 (1–3)	-	-
SSDDI	2 (2–3)	-	-

Abbreviations: SBP: Systolic blood pressure; DBP: Diastolic blood pressure; FRS: Framingham Risk Score for Coronary Heart Disease; ESSDAI: EULAR Primary Sjögren’s Syndrome Disease Activity Index; SSDDI: Sjögren’s Syndrome Disease Damage Index. Data are presented as whole number (percentage), mean ± standard deviation or median (IQR) * chi-square test (Fisher’s exact test), *t*-test for independent samples or Mann-Whitney U test, ^†^ time period from the initial diagnosis.

**Table 2:** Laboratory parameters of pSS patients and healthy controls.

Parameter	pSS Group (N = 52)	Control Group (N = 52)	p *
Erythrocytes (×10^12^/L)	4.37 ± 0.42	4.52 ± 0.32	0.047
Hemoglobin (g/L)	130.5 ± 11.0	138.4 ± 11.5	<0.001
MCV (fL)	87.1 ± 12.8	93.1 ± 16.1	0.034
Leukocytes (×10^12^/L)	4.88 ± 1.38	5.94 ± 1.81	0.001
Platelets (×10^9^/L)	230.8 ± 52.1	244.1 ± 47.9	0.179
Urea (mmoL/L)	5.51 ± 1.65	5.47 ± 1.44	0.915
Creatinine (mmoL/L)	65.9 ± 18.5	63.1 ± 14.6	0.378
ESR (mm/h)	16.5 (8.0–27.5)	10.0 (6.0–16.0)	0.006
hsCRP (mg/L)	1.30 (0.8–3.8)	1.15 (0.7–3.3)	0.767
TC (mmoL/L)	5.60 ± 1.12	6.13 ± 0.93	0.010
LDL (mmoL/L)	3.56 ± 0.96	3.91 ± 1.15	0.093
TG (mmoL/L)	1.18 ± 0.42	1.42 ± 0.85	0.072
HDL (mmoL/L)	1.52 ± 0.37	1.57 ± 0.32	0.436
C3 (g/L)	1.21 ± 0.22	1.19 ± 0.17	0.567
C4 (g/L)	0.26 ± 0.19	0.31 ± 0.09	0.197
Anti SSB/La (AU/mL)	33.5 (2.5–81.5)	-	-
Anti SSA/Ro52 (AU/mL)	91.0 (16.5–162.0)	-	-
Anti SSA/Ro60 (AU/mL)	103.0 (56.5–117.0)	-	-

Abbreviations: MCV: Mean corpuscular volume; ESR: Erythrocyte sedimentation rate; hsCRP: high sensitivity C-reactive protein; TC: total cholesterol; LDL: low density lipoprotein; TG: triglycerides; HDL: high density lipoprotein; C3: complement component C3; C4: complement component C4; Data are presented as whole number (percentage), mean ± standard deviation or median (IQR), * *t*-test for independent samples or Mann-Whitney U test.

**Table 3:** Characteristics of primary Sjögren’s syndrome patients based on adropin level.

Parameter	Lower Adropin (<3.73 ng/mL) (N = 26)	Higher Adropin (>3.73 ng/mL) (N = 26)	*p* *
Age (years)	51.0 ± 9.83	57.6 ± 12.3	0.284
Body mass index (kg/m^2^)	24.8 ± 4.1	24.83 ± 2.5	0.978
SBP (mmHg)	123.6 ± 18.46	128.8 ± 14.9	0.270
DBP (mmHg)	79.6 ± 8.8	80.8 ± 5.9	0.086
Total cholesterol (mmoL/L)	5.56 ± 1.08	5.76 ± 1.21	0.535
HDL (mmoL/L)	1.46 ± 0.36	1.56 ± 0.38	0.362
LDL (mmoL/L)	3.35 ± 0.89	3.76 ± 0.99	0.119
TG (μmol/L)	1.15 ± 0.43	1.20 ± 0.40	0.694
hsCRP (mg/L)	1.35 (0.9–2.7)	1.15 (0.80–5.6)	0.869
Anti SSB/La (AU/mL)	36.0 (7.0–79.0)	9.5 (2.0–88.0)	0.734
AntiSSA/Ro52 (AU/mL)	73.0 (12.0–125.0)	109.0 (17.0–172.0)	0.272
Anti SSA/Ro60 (AU/mL)	101.0 (60.0–116.0)	107.5 (52.0–118.0)	0.963
Disease duration (years) ^†^	6 (4.0–12.0)	6 (3.0–10.0)	0.607
ESSDAI	2.0 (1.0–3.0)	2.5 (1.0–3.0)	0.641
SSDDI	3.0 (2.0–4.0)	2.0 (1.0–2.0)	<0.001
FRS (%)	4.25 (1.2–6.2)	2.3 (1.4–5.2)	0.374

Abbreviations: SBP: systolic blood pressure; DBP: diastolic blood pressure; HDL: high density lipoprotein; LDL: low density lipoprotein; TG: Triglycerides; hsCRP: high sensitivity C-reactive protein; ESSDAI: EULAR Primary Sjögren’s Syndrome Disease Activity Index; SSDDI: Sjögren’s Syndrome Disease Damage Index; FRS: Framingham Risk Score for Coronary Heart Disease; Data are presented as whole number (percentage), mean ± standard deviation or median (IQR), * *t*-test for independent samples or Mann-Whitney U test, ^†^ time period from the initial diagnosis.

**Table 4:** Correlation analysis between serum adropin levels and different biochemical and anthropometric parameters.

Parameter	r *	*p*
Age (years)	−0.133	0.345
Body mass index (kg/m^2^)	−0.133	0.348
hsCRP (mg/L)	0.158 ^†^	0.262
TC (mmoL/L)	0.224	0.109
TG (mmoL/L)	−0.083	0.557
HDL (mmoL/L)	0.290	0.036
LDL (mmoL/L)	0.178	0.206
SBP (mmHg)	0.118	0.404
DBP (mmHg)	0.155	0.271
Anti SSA/Ro60 (AU/mL)	0.123 ^†^	0.385
Anti SSA/Ro52 (AU/mL)	0.307 ^†^	0.026
Anti SSB/La (AU/mL)	−0.009 ^†^	0.946
ESSDAI	0.051 ^†^	0.721
SSDDI	−0.401 ^†^	0.003
Disease duration (years)	−0.041 ^†^	0.770

Abbreviations: hsCRP: high sensitivity C-reactive protein; TC: Total cholesterol; TG: triglycerides; HDL: high density lipoprotein; LDL: low density lipoprotein; SBP: systolic blood pressure; DBP: diastolic blood pressure; ESSDAI: EULAR Primary Sjögren’s Syndrome Disease Activity Index; SSDDI: Sjögren’s Syndrome Disease Damage Index, * Pearson’s correlation coefficient, ^†^ Spearman’s rank correlation coefficient.

**Table 5:** Multiple Linear Regression model of independent predictors for serum adropin levels.

Variable	β ^1^	SE ^2^	*t*-Value	*p*
Age (years)	−0.013	0.011	−1.134	0.263
Sex	0.220	0.367	0.602	0.550
BMI (kg/m^2^)	−0.007	0.002	−0.268	0.794
Anti SSA/Ro52	0.001	0.001	1.551	0.128
HDL	0.903	0.283	3.191	0.002
FRS	0.025	0.032	0.768	0.447
Disease duration ^†^	0.006	0.015	0.394	0.695
ESSDAI	0.044	0.061	0.718	0.476
SSDDI	−0.202	0.073	−2.754	0.008

Abbreviations: BMI: body mass index; HDL: high density lipoprotein; FRS: Framingham Risk Score for Coronary Heart Disease; ESSDAI: EULAR Primary Sjögren’s Syndrome Disease Activity Index; SSDDI: Sjögren’s Syndrome Disease Damage Index, ^1^ unstandardized coefficient β, ^2^ standard error, ^†^ time period from the initial diagnosis.

## Data Availability

The data presented in this study are available on request from the corresponding author. The data are not publicly available because some of the data set will be used for further research.

## References

[1] Patel R., Shahane A. (2014). The epidemiology of Sjögren’s syndrome. Clin. Epidemiol..

[2] Pérez-De-Lis M., Akasbi M., Sisó A., Diez-Cascon P., Brito-Zerón P., Diaz-Lagares C., Ortiz J., Perez-Alvarez R., Ramos-Casals M., Coca A. (2010). Cardiovascular risk factors in primary Sjögren’s syndrome: A case-control study in 624 patients. Lupus.

[3] Horvath I.F., Szanto A., Papp G., Zeher M. (2014). Clinical course, prognosis, and cause of death in primary Sjögren’s syndrome. J. Immunol. Res..

[4] Vaudo G., Bocci E.B., Shoenfeld Y., Schillaci G., Wu R. (2005). Precocious intima-media thickening in patients with primary Sjogren’s syndrome. Arthritis Rheum..

[5] Atzeni F., Sarzi-Puttini P., Signorello M.C., Gianturco L., Stella D. (2014). New parameters for identifying subclinical atherosclerosis in patients with primary Sjogren’s syndrome: A pilot study. Clin. Exp. Rheumatol..

[6] Akyel A., Tavil Y., Yayla C., Tufan A., Kaya A. (2012). Endothelial dysfunction in primary Sjogren syndrome. West Indian Med. J..

[7] Melissaropoulos K., Bogdanos D., Dimitroulas T., Sakkas L.I., Kitas G.D., Daoussis D. (2020). Primary Sjögren’s Syndrome and Cardiovascular Disease. Curr. Vasc. Pharmacol..

[8] Li L., Xie W., Zheng X.L., Yin W.D., Tang C.K. (2016). A novel peptide adropin in cardiovascular diseases. Clin. Chim. Acta.

[9] Yosaee S., Soltani S., Sekhavati E., Jazayeri S. (2016). Adropin- A Novel Biomarker of Heart Disease: A Systematic Review Article. Iran. J. Public Health.

[10] Kumar K.G., Trevaskis J.L., Lam D.D., Sutton G.M., Koza R.A., Chouljenko V.N., Kousoulas K.G., Rogers P.M., Kesterson R.A., Thearle M. (2008). Identification of adropin as a secreted factor linking dietary macronutrient intake with energy homeostasis and lipid metabolism. Cell Metab..

[11] Lovren F., Pan Y., Quan A., Singh K.K., Shukla P.C., Gupta M., Al-Omran M., Teoh H., Verma S. (2010). Adropin is a novel regulator of endothelial function. Circulation.

[12] Petersen T.N., Brunak S., von Heijne G., Nielsen H. (2011). SignalP 4.0: Discriminating signal peptides from transmembrane regions. Nat. Methods.

[13] Niepolski L., Grzegorzewska A.E. (2016). Salusins and adropin: New peptides potentially involved in lipid metabolism and atherosclerosis. Adv. Med. Sci..

[14] Zhao L.P., You T., Chan S.P., Chen J.C., Xu W.T. (2016). Adropin is associated with hyperhomocysteine and coronary atherosclerosis. Exp. Ther. Med..

[15] Wu L., Fang J., Chen L., Zhao Z., Luo Y., Lin C., Fan L. (2014). Low serum adropin is associated with coronary atherosclerosis in type 2 diabetic and non-diabetic patients. Clin. Chem. Lab Med..

[16] Vitali C., Bombardieri S., Jonsson R., Moutsopoulos H.M., Alexander E.L., Carsons S.E., Daniels T.E., Fox P.C., Fox R.I., Kassan S.S. (2002). European Study Group on Classification Criteria for Sjögren’s Syndrome. Classification criteria for Sjögren’s syndrome: A revised version of the European criteria proposed by the American-European Consensus Group. Ann. Rheum. Dis..

[17] Wilson P.W., D’Agostino R.B., Levy D., Belanger A.M., Silbershatz H. (1998). Prediction of coronary heart disease using risk factor categories. Circulation.

[18] Seror R., Ravaud P., Bowman S.J., Baron G., Tzioufas A., Theander E. (2010). EULAR Sjögren’s syndrome disease activity index: Development of a consensus systemic disease activity index for primary Sjögren’s syndrome. Ann. Rheum. Dis..

[19] Vitali C., Palombi G., Baldini C., Benucci M., Bombardieri S., Covelli M., Del Papa N., De Vita S., Epis O., Franceschini F. (2007). Sjögren’s Syndrome Disease Damage Index and disease activity index: Scoring systems for the assessment of disease damage and disease activity in Sjögren’s syndrome, derived from an analysis of a cohort of Italian patients. Arthritis Rheum..

[20] Verma S., Buchanan M.R., Anderson T.J. (2003). Endothelial function testing as a biomarker of vascular disease. Circulation.

[21] Celik A., Balin M., Kobat M.A., Erdem K., Baydas A., Bulut M., Altas Y., Aydin S., Aydin S. (2013). Deficiency of a new protein associated with cardiac syndrome X; called adropin. Cardiovasc. Ther..

[22] Sato K., Yamashita T., Shirai R., Shibata K., Okano T., Yamaguchi M., Mori Y., Hirano T., Watanabe T. (2018). Adropin Contributes to Anti-Atherosclerosis by Suppressing Monocyte-Endothelial Cell Adhesion and Smooth Muscle Cell Proliferation. Int. J. Mol. Sci..

[23] Wanchu A., Khullar M., Sud A., Bambery P. (2000). Elevated nitric oxide production in patients with primary Sjögren’s syndrome. Clin. Rheumatol..

[24] Couffinhal T., Duplàa C., Moreau C., Lamazière J.M., Bonnet J. (1994). Regulation of vascular cell adhesion molecule-1 and intercellular adhesion molecule-1 in human vascular smooth muscle cells. Circ. Res..

[25] Lee H.M., Kim H.J., Won K.J., Choi W.S., Park S.H., Song H., Park P.J., Park T.K., Lee C.K., Kim B. (2008). Soluble form of vascular cell adhesion molecule 1 induces migration and proliferation of vascular smooth muscle cells. J. Vasc. Res..

[26] Konttinen Y.T., Platts L.A., Tuominen S., Eklund K.K., Santavirta N., Törnwall J., Sorsa T., Hukkanen M., Polak J.M. (1997). Role of nitric oxide in Sjögren’s syndrome. Arthritis Rheum..

[27] Yu H.Y., Zhao P., Wu M.C., Liu J., Yin W. (2014). Serum adropin levels are decreased in patients with acute myocardial infarction. Regul. Pept..

[28] Ignarro L.J., Cirino G., Casini A., Napoli C. (1999). Nitric oxide as a signaling molecule in the vascular system: An overview. J Cardiovasc. Pharmacol..

[29] Akcilar R., Kocak F.E., Simsek H., Akcilar A., Bayat Z., Ece E., Kokdasgil H. (2016). Antidiabetic and hypolipidemic effects of adropinin streoptozotocin-induced type 2 diabetic rats. Bratisl. Lek. Listy.

[30] Akcılar R., Emel Koçak F., Şimşek H. (2016). The effect of adropin on lipid and glucose metabolism in rats with hyperlipidemia. Iran. J. Basic Med. Sci..

[31] Yolbas S., Kara M., Kalayci M., Yildirim A., Gundogdu B., Aydin S., Koca S.S. (2018). ENHO gene expression and serum adropin level in rheumatoid arthritis and systemic lupus erythematosus. Adv. Clin. Exp. Med..

[32] Yolbas S., Kara M., Yilmaz M., Aydin S., Koca S.S. (2016). Serum adropin level and ENHO gene expression in systemic sclerosis. Clin. Rheumatol..

[33] Bozic J., Borovac J.A., Galic T., Kurir T.T., Supe-Domic D., Dogas Z. (2018). Adropin and Inflammation Biomarker Levels in Male Patients with Obstructive Sleep Apnea: A Link With Glucose Metabolism and Sleep Parameters. J. Clin. Sleep Med..

[34] Brnić D., Martinovic D., Zivkovic P.M., Tokic D., Tadin Hadjina I., Rusic D., Vilovic M., Supe-Domic D., Tonkic A., Bozic J. (2020). Serum adropin levels are reduced in patients with inflammatory bowel diseases. Sci. Rep..

[35] Kuliczkowska-Płaksej J., Mierzwicka A., Jończyk M., Stachowska B., Urbanovych A., Bolanowski M. (2019). Adropin in women with polycystic ovary syndrome. Endokrynol. Pol..

[36] Tičinović Kurir T., Miličević T., Novak A., Vilović M., Božić J. (2020). Adropin—Potential Link in Cardiovascular Protection for Obese Male Type 2 Diabetes Mellitus Patients Treated with Liraglutide. Acta Clin. Croat..

[37] Retamozo S., Akasbi M., Brito-Zerón P., Bosch X., Bove A., Perez-de-Lis M., Jimenez I., Soto-Cardenas M.J., Gandia M., Diaz-Lagares C. (2012). Anti-Ro52 antibody testing influences the classification and clinical characterisation of primary Sjögren’s syndrome. Clin. Exp. Rheumatol..

[38] Bartoloni E., Baldini C., Schillaci G., Quartuccio L., Priori R., Carubbi F., Bini V., Alunno A., Bombardieri S., De Vita S. (2015). Cardiovascular disease risk burden in primary Sjögren’s syndrome: Results of a population-based multicentre cohort study. J. Intern. Med..

[39] Salmon J.E., Roman M.J. (2008). Subclinical atherosclerosis in rheumatoid arthritis and systemic lupus erythematosus. Am. J. Med..

[40] Shoenfeld Y., Gerli R., Doria A., Matsuura E., Cerinic M.M., Ronda N., Jara L.J., Abu-Shakra M., Meroni P.L., Sherer Y. (2005). Accelerated atherosclerosis in autoimmune rheumatic diseases. Circulation.

[41] Sabio J.M., Sánchez-Berná I., Martinez-Bordonado J., Vargas-Hitos J.A., Navarrete-Navarrete N., Expósito Ruíz M., Jiménez-Alonso J. (2015). Prevalence of and factors associated with increased arterial stiffness in patients with primary Sjögren’s syndrome. Arthritis Care Res..

[42] Gravani F., Papadaki I., Antypa E., Nezos A., Masselou K., Ioakeimidis D., Koutsilieris M., Moutsopoulos H.M., Mavragani C.P. (2015). Subclinical atherosclerosis and impaired bone health in patients with primary Sjogren’s syndrome: Prevalence, clinical and laboratory associations. Arthritis Res. Ther..

[43] Gu X., Li H., Zhu X., Gu H., Chen J., Wang L., Harding P., Xu W. (2015). Inverse Correlation Between Plasma Adropin and ET-1 Levels in Essential Hypertension: A Cross-Sectional Study. Medicine.

[44] Gulen B., Eken C., Kucukdagli O.T., Serinken M., Kocyigit A., Kılıc E., Uyarel H. (2016). Adropin levels and target organ damage secondary to high blood pressure in the ED. Am. J. Emerg. Med..

[45] Çelik H.T., Akkaya N., Erdamar H., Gok S., Kazanci F., Demircelik B., Cakmak M., Yigitoglu R. (2015). The Effects of Valsartan and Amlodipine on the Levels of Irisin, Adropin, and Perilipin. Clin. Lab.

[46] Boric-Skaro D., Mizdrak M., Luketin M., Martinovic D., Tokic D., Vilovic M., Supe-Domic D., Kurir T.T., Bozic J. (2021). Serum Adropin Levels in Patients on Hemodialysis. Life.

[47] Aydin S. (2014). Three new players in energy regulation: Preptin, adropin and irisin. Peptides.

[48] Ganesh Kumar K., Zhang J., Gao S., Rossi J., McGuinness O.P., Halem H.H., Culler M.D., Mynatt R.L., Butler A.A. (2012). Adropin deficiency is associated with increased adiposity and insulin resistance. Obesity.

